# Topics of Public Interest

**Published:** 1916-12

**Authors:** 


### TOPICS OF PUBLIC INTEREST

---

**Proposed Uniform Legislation Regarding Traffic in N. Y. State.** In accordance with action taken at the conference of mayors and other city officials, a tentative draft of a law has been made, which is now submitted to the various cities of the state for endorsement. This is too long to publish in full but it may be said, in general, that it is a collection of existing laws and customs, with the significant exception that regulations that have been shown by experience to be unwise are omitted or rescinded, and that the general principle is established that local governments may not tax or make unreasonable and peculiar limitations to the use of the public highways by the public. As a whole, the proposed state law is sound, reasonable and fair, and will undoubtedly be copied in its essential features by other states. Being still subject to scrutiny, we take the liberty to offer the following suggestions:

Section 22. A right hand signal is required of the driver of an automobile before reducing speed or stopping. This is obviously impracticable and even dangerous for left-hand drive cars, now in the vast majority. In congested districts any signalling by hand is often impossible, so long as automobiles are constructed as at present and so long as human beings continue to be limited to two hands. It is rather implied elsewhere that the driver of a car in the rear of another in the congested district shall act independently of such signals, but the law should be explicit on this point. Hand signalling for right hand turns should not be required at all in cities, as following vehicles do not need them if they observe ordinary traffic rules, and as pedestrians are better warned by sound of horn, etc.

Preliminary definitions. A district in which dwelling houses average "less than 200 feet between them for a quarter of a mile" is scarcely a congested district, and the tendency of small hamlets to emphasize their importance should be curbed rather than encouraged. In this connection it may be suggested that, as many villages and cities have included much open country in their boundaries, Thank You signs should be placed where the actual urban or village population begins, as indicated by pavements, sidewalks and the occupation of, say 25-50% of the frontage by buildings.

Section 56. "No vehicle shall remain stationary on any street, avenue or public place for a longer period than four hours." As a general rule, this is proper enough, but a law ought to be enforced or changed. A good many social functions last more than four hours, so do labor cases, and a physician, expecting an emergency call of any kind ought not to be compelled to get up and move his car in the night, unless he has to do so to meet the expected engagement. Again, strangers coming to town on business, are often prevented from returning to their cars within this time limit.

Section 66. "Disabled vehicles shall not be left on the street longer than three hours." This again is sound on general principles but, in a great many special cases, it is striking a man when he is down. An exception should certainly be made of the night hours. Merely waiting till day often means a comparatively simple and cheap adjustment or repair instead of a heavy mechanic's bill. For electric vehicles, it means running on a recuperated battery instead of being towed. In the writer's experience, police officers have been uniformly considerate and reasonable in regard to crippled cars, and the law should not prevent them from continuing to be. The law might reasonably require the owner or operator of a crippled car to report the case by telephone, unless himself so much damaged as to be unable to do so, and it might further require the lighting of the car, including the attachment of lanterns by the police and a reasonable charge for the service, when the lights of the car itself cannot be operated.

Section 91. This requires attention to all signs posted to direct traffic in any way. This is, on general principles, wise, but it must be remembered that drivers have only two eyes, must see in 120° of arc at all times and in 360° often. It is impossible that the most careful and conscientious driver shall see every sign posted and due allowance should be made for this fact. If this provision stands, it should be accompanied by the further provision that advertisements and other non-pertinent notices should be excluded from positions along streets and roads where traffic directions would naturally be looked for. Drivers are already annoyed by slowing down or stopping to secure information as to directions and distance, and finding that they are instead being informed as to what cigars they should smoke, or that a school tax is due or that a farmer is going to auction off his stock.

Section 95, defining the powers left to local authorities to frame ordinances to provide for special local conditions, should be modified so as to secure the endorsement of some general board before any such ordinance becomes effective.

As stated, the proposed law is excellent. It is not only a good traffic law, but it establishes, or at least confirms two important general precedents—the limitations of local and arbitrary legislation in the interests of a class, the resident as opposed to the non-resident population, and the principle that the pedestrian has responsibilities as well as privileges. It is to be hoped that the tentative provisions may be carefully criticised so that the law as passed shall be as near perfection and permanence as is humanly possible.

---

**Narcotic Laws.** Practically all states and dependencies of the U. S. have some form of narcotic law, as well as a few cities. The Alaska law applies only to opium; those of La., Miss., Pa., S. C. seem to apply to cocaine only. N. Y. and Va. also require prescriptions for hypodermic syringes. Prescriptions for narcotic drugs are required by practically all. Nev. allows dispensing only by pharmacists, otherwise physicians are either allowed to dispense or "administer" under various restrictions. Ariz., Calif., Fla., Me., Mass., Minn., Utah and Vt. require a record to be kept. Col., Conn., Ill. require a record unless the narcotic is administered during personal attendance. S. D. and Tenn. require a duplicate of the prescription to be kept. Conn. and Mass. (and N. Y.?) specify amounts not requiring record. Me. and N. D. prohibit administration to addicts. Miss. allows administration to addicts only for immediate preservation of life. Strangely enough, the refilling of prescriptions is prohibited only by Conn., Fla., Ill., Mass., and Neb. (?). Possession of a narcotic by the laity is prohibited in most states. Some special requirements are as follows: Ariz. requires consultation and report to local health officer after narcotics have been prescribed for 3 weeks. Calif. requires the reporting of names of addicts within 24 hours to Board of Pharmacy and provides for commitment of addicts. Delaware requires that narcotics must be labeled and must be sold only to the sick. Fla. requires that reports of narcotics be sent to local health officer within 3 days and that addicts refusing treatment be reported to local health officer or county judge. Incurables, after investigation, by board of health or judge, may have special permit. Idaho provides for revocation of license if the law is broken. Ia. includes cocain, cotton root, ergot, tansy and savin. The Kas. law applies to morphine, cocaine and chloral. Nev. requires copies of all orders for narcotics to be mailed to the board of pharmacy within 24 hours. Okla. provides for teaching the effects of narcotics in the public schools. Ore. holds it unlawful for any person to manufacture, sell or dispense any drugs unless he is a registered pharmacist. (Condensation of table in Medical World, Nov.).

It would seem, inasmuch as the national law regarding narcotics has got around the constitution by adding a tax on physicians, that all states might unite on this law, provided that it be modified in accordance with common sense, and interpreted by authorities having some rudimentary knowledge of drugs. The inclusion of apomorphine and the ruling that personal attendance does not mean what it says except under special circumstances, as well as numerous other inconsistencies to which we have called attention from time to time, shows that this provision is not a figure of speech. The state laws mentioned afford valuable suggestions as to amendment, some by very wise provisions, some by very foolish ones, as the obvious attempt in the interests of druggists to exclude physicians from the proper use of drugs by personal dispensing.

---

**A Modern Solomon.** The Illinois Medical Journal narrates the case of a man aged 65 convicted of criminal assault on a young girl. As he was in poor health (?), the court felt that the usual prison term would amount to imprisonment for life and therefore gave the alternative of sterilization and freedom, which was accepted. Here we have an elderly degenerate, relieved of all anxiety as to offspring, turned loose on society, with a warning to observe certain age limits. The press reports implied that the judge thought that sterilization meant immunity from venereal disease. Balzac has a story that begins in the same way but in which the accused is turned loose from the gallows by demonstrating the preservation of his sexual power to an advanced age. It is a question whether the mediaeval or modern "justice" was the more rational.

---

**The Medical Corps of the U. S. Navy** has been increased by 300 by recent legislation.

---

**The Mann Law** is before the U. S. Supreme Court for argument regarding its applicability to voluntary trips for immoral purposes, where the element of white slavery or undue suasion of young girls is absent. It has already been shown that this act is a great assistance to black mailers and, at the beginning of the discussion, we called attention to the danger of legislation on matters of pure ethics and morality, not involving damage to person or property rights. Something must be left to the individual conscience if we wish to develop a sense of personal responsibility and legislation on matters of abstract right and wrong comes dangerously near the line of personal and religious liberty.

---

**War Losses.** Frank H. Simmonds (N. Y. Tribune, Oct. 22) estimates the war losses as follows: As a matter of interest we give comparative estimates by newspapers, up to May 31, 1915.

| | 26 months. | 10 months. |
|---|---|---|
| France | 2,500,000 | 1,300,000 |
| Russia | 5,750,000 | 3,780,000 |
| Great Britain | 1,400,000 | 471,000 |
| Italy | 350,000 | ........ |
| Belgium | ....... | 113,000 |
| Total Allies | 10,000,000 | 5,664,000 |
| Germany | 4,000,000 | 4,000,000 |
| Austria | 4,000,000 | 4,385,000 |
| Turkey | ....... | 349,000 |
| Total, Central Powers | 8,000,000 | 8,734,000 |

Obviously, the earlier casualties were exaggerated, though perhaps by failure to consider the fact that nearly 90% of the non-fatally wounded are restored to service. English tabulations of official German reports show a total loss of 3,755,692 for the first 27 months of the war. Of these 910,234 were deaths. Allowing for delayed reports, especially of missing and prisoners, Simmonds' estimate seems conservative. He calculates that the central powers have lost about 1/3 of their man-power and the allies about 1/6. But, each year, for quite a while to come, about 1/10 of the 10% of military strength of a nation, will be compensated by the rising generation.

---

**Smuggled and Fraudulent Neosalvarsan.** A Federal grand jury has indicted Dr. Jean F. Strandgaard of Toronto and a steward on the steamer United States both for smuggling neosalvarsan and for preparing a worthless substitute. During July, Strandgaard had 15,000 ampoules blown in Jersey City and filled with starch or salt. Since then, clever imitations of German and especially English packages have been peddled by women in N. Y., Chicago and various cities of the middle west and south. Physicians are warned of the danger of purchasing these drugs of unreliable persons and are requested to inform Chief Inspector E. R. Norwood of the Customs Service in N. Y. City of attempts to peddle such wares.

---

**Increase in Medical Corps U. S. Army. Transfers From Reserve Corps.** The Hay bill, effective June 3, 1916, provides for a total of 1553 medical officers, the present strength being only 336. 336 officers of the Medical Reserve Corps are on active duty, to make up for the temporary shortage. Mr. Gandy has introduced a bill in the House, providing for the transfer of members of the Reserve Corps to the Medical Corps of the Army, with promotion to the rank of captain, to take relative rank according to their length of service, but below captains in the regular medical corps. This transfer and promotion is irrespective of age but subject to examination, satisfactory service and five years' military service, including that as contract, or volunteer surgeon or under enlistment. It is proposed also to allow as an alternative, four years' in the Public Health Service. This bill and the amendment should be approved. Indeed, under the present circumstances, it would seem wise to admit to the Army Medical Corps, physicians who have had reasonable experience in almost any kind of military or government service, subject to such examinations as might establish a reasonable degree of proficiency. The sudden, relatively enormous increase in the nominal strength of the medical corps, amounts to nearly half of the total number of medical graduates for any one of the last few years and probably exceeds the number of men available according to ordinary standards of appointment. The requirements as to post-graduate hospital experience on the one hand and as to age on the other, practically limits eligibility to the classes from 1910 to 1914. It seems doubtful whether 1100 men could be found in this total who would be desirous of army service and who would meet the physical and intellectual requirements established by precedent. If they could be found, it would be highly undesirable to have approximately 3 quarters of the army medical corps in so nearly the same age group as this would block promotions for many years to come and would then suddenly call for very rapid promotions and a large number of new appointments. By filling the present sudden increase in the personnel of the corps with men of moderate military experience and of widely different age, many embarrassing problems both of the present and the future would be obviated.

---

**The High Cost of Living.** The increase in the price of milk is ascribed to demands on the part of the ultimate producer, explained as necessary because of demands by the ultimate consumer through the various health departments for a rich and clean product. In this connection, it is interesting to note the complaint of a milk producer that the dealers were not willing to split "50-50" with him but wanted at least 60% of the advance. It is evident that milk produced under modern conditions can not be so cheap as under those formerly in vogue and that milk of full nutritive value and insured so far as possible against tubercular infection, manure bacteria and ordinary germs of fermentation, is worth more than the former city milk. But the main element in increased cost of foods is that everyone wants more for his day's work than formerly, and a great deal more for his hour's work. We can't get away from the fact that the unit of labor is the ultimate unit of value. In some instances, labor may be systematized and aided by machinery so as to permit a cheaper monetary cost in spite of high wages but this does not apply to food industries in general where direct human labor remains the most important factor. Let us not forget that, in proportion to its necessity, food costs less in terms of average labor of the consumer, than ever before. Present exceptions are largely due to disturbances of supply and demand due to the war and, in more cases than we realize, the present high prices are lower than those prevalent 50 years ago, and probably only temporary. Tea, coffee, sugar and flour are illustrations.

---

**The National Board of Medical Examiners,** through the Secretary, J. S. Rodman of Philadelphia, report that the first examination was held Oct. 16-21 at Washington. 32 applications were received from 17 states and 24 medical schools. 16 were accepted as having qualified for examination. 10 took the examination and 5 passed: Harry S. Newcomer and Wm. White Southard of Johns Hopkins, Orlow C. Snyder of the University of Michigan, Thos. A. Johnson and Hjorleifur T. Kristjanson of Rush. The next examination will be held in Washington in June. Application should be made to the Secretary. (Note: While the policy of this journal has always been in favor of high educational requirements, it occurs to us that these statistics indicate a much higher demand than can be inforced as a practical requirement for license and that if the National Board is to acquire official recognition and ultimately supplant the state system of licensure, it must adhere to a lower standard. These figures are about what would be expected from an examining board for the army, navy or public health service).

---

**The Dept. of Arts and Science, University of Buffalo** announces a course of lectures, Mondays, 5 p. m. in Townsend Hall, Niagara Square, to which the public is invited free. Frank H. Severance, L.H.D., Secretary of the Buffalo Historical Society, will give a series of lectures on the History of the Niagara Region, Nov. 13-Dec. 4, Chas. E. Rhodes, M. A., will lecture Dec. 11 on Rambles in the Lake District.

---

**U. S. Civil Service Examinations** will be held Dec. 13, at various places as determined by the residences of applicants, as follows: Physicians, Indian Service, $1,000-$1,200. Panama Service, $1,800. Dental Interne, St. Elizabeth's Hospital, Washington, $600. Those wishing to take the examinations should apply to the Secretary at Washington, for details as to requirements, etc.

---

**Smoke Consumption.** Osborn Monnett addressed the Greater Buffalo Club Nov.. 22, on the Smoke Nuisance. He advocated the use of powdered coal, crude oil, and proper stoking. Smoke eliminating apparatus for a locomotive could be installed for $75.

---

**The Alvarenga Prize** amounting to $250 will be awarded by the College of Physicians of Philadelphia, July 14, 1917. Essays may be upon any subject in medicine, must not have been already published, must be type-written in English and must be in the hands of the Secretary, Dr. Francis R. Packard, 19 S. 22 St., Philadelphia, by May 1, 1917.

---

**Paratyphoid Carriers.** The 14th Regiment had 130 cases and furnished 40 healthy carriers; the 71st, 14 cases, 5 carriers.

---

**Lebanon Hospital, New York City,** Surgical Clinics, Wednesdays, at 3 o'clock, held by Dr. Parker Syms and Dr. M. R. Bookman, Nov. 1 to Mar. 1. Take Subway West Farms Division to Jackson Ave. Station. See Daily Bulletin, Academy of Medicine.

---

**Buffalo Public School Registration.** For 1915-16, the registration was 51,636 for 1916-17, 52,185 in grammar schools. In high schools the respective figures were 5,641 and 5,469. It should be noted that these statistics show a relative decrease with regard to normal change of population and an actual decrease for high schools. It is explained that the scarcity of labor and resulting high wages explain the diminution.

---

**Taxation of Visiting Automobiles.** The U. S. Supreme Court will shortly consider a test case, based on the requirement of a state license by New Jersey—which is simply one example selected—and the decision will bear on the constitutionality of a bill introduced into congress which provides that the payment of a tax in one state shall relieve the owner of any further obligation to take out state licenses. There is ample precedent that any state should accord the courtesy of the use of its highways to a visitor from another state who has complied with local laws. So far as we know, no state has ever attempted to tax a visitor on foot for the use of sidewalks nor to tax for the use of roads, any vehicle except bicycles, automobiles and motorcycles. Tolls will probably not be affected by the decision but it may be pointed out that in the more enlightened states, there is a strong tendency toward doing away with tolls of all kinds. Physicians of Washington who are at present practically compelled to carry licenses for the D. C., Va. and Md., will be especially interested in the decision. In passing it may be remarked that tours southward to Washington cost about as much for tolls as for gasoline and that the delays in paying toll aggregate the average for punctures and blow-outs.

---

**Endowment of Medical School of University of Chicago.** The Rockefeller Foundation has appropriated $2,000,000, the University will appropriate at least as much beside furnishing a site worth $500,000 and it is expected that about $3,500,000 will be raised by subscription, making a total of $8,000,000. Good, but—suppose the $2,000,000 and whatever could be raised by popular subscription stimulated by a gift from without, were distributed among about 50 medical schools that fill local needs, that are conscientiously trying to meet increasing requirements, and that are having a hard struggle for existence?

---

**The Presidential Election.** It would not be proper to consider this subject from the ordinary political standpoint but there are certain psychologic factors of interest. The closeness of the vote in many states and the unexpected political changes in others indicates a growing tendency to independent thought, regardless of political affiliation and to change of mind to meet changed conditions. This is a wholesome tendency, however one regards the wisdom of the majority. At the risk of venturing too close to actual politics, it may even be said that a great many voters refused to blame the president for changing his mind under altered conditions, or for making what they might have regarded as mistakes under circumstances that rendered judgment based on the past and fully providing for the future, exceedingly difficult. On the other hand, they demanded a detailed and cricumstantial criticism from his opponent. In other words, the people assumed the judgment to decide as if they themselves were the government and this attitude, as well as the recognition of the unfairness of an electoral vote contrary to the popular vote may ultimately do away entirely with the electoral college which has never been more than a theory since the first few presidential elections. The fact that, in the present instance, the aggregate electoral vote will fairly represent the aggregate popular vote, perhaps will further such a demand as in itself unbiased by political results. The conspicuous differences in the majorities of various candidates on the same ticket, also shows that the old idea of party loyalty is losing ground. The vote for prohibition in certain states is also significant. Perhaps most interesting of all is the vote by women. There was comparatively little to justify either the aspiration or the fear that women would vote as a sex. Speaking broadly, the women's vote corresponded closely with the male vote, yet with sufficient local fluctuations to indicate personal independence of judgment. It cannot be said either that women have voted as a unit for an individual or a policy that the sex might be considered to favor, neither can it be said that they have simply been influenced by the male members of their families. In short, the suffrage must be regarded as an ultimate justice rather than a rapid method of purifying politics and accomplishing reforms or, on the other hand, as something to which the obvious expense in money and effort required to handle a double number of voters oppose practical objections not offset by any notable improvement of the exercise of the franchise. The decision, in states not already granting the suffrage to women, or assumed by the country as a whole, will probably depend to some degree upon how the women who are already voters deal with the vast amount of legislation establishing privileges for the sex on the old theory that they were not competent to discharge the entire duty of citizens and must therefore be specially protected and provided for.

---

**Roosters, Dogs, Gas Stoves Without Chimneys and Milk Standards** are engaging the attention of the Buffalo Dept. of Health. Ordinances are sought which will effectually control these matters, in accordance with general principles of the code. Much popular objection has been manifested to too stringent legislation. The keeping of chickens should be encouraged for its influence on food prices, provided a fair amount of space is available. Roosters can be quieted by section of the vocal cords—a simple and probably painless operation in birds—and by various covers, throat rings, etc. The average dog is quieter, more trustworthy and less likely to inflict bites than the average human being. Being subject to license, his personal merits and demerits are subject to formal proof. We have little sympathy for nervous cranks who want everyone and everything to keep still while they sleep and, on the other hand, we are all annoyed by unnecessary noises depending on carelessness and lack of consideration. We don't like to think of even a few people killed by loose gas connections on stoves in rooming houses and, on the other hand we don't want to suffer from cold on account of temporary lack of gas pressure or because the furnace man has made a slip, when we have a perfectly good gas heater that can be attached anywhere but for which a chimney cannot be built in every room. Somehow, it ought to be possible to frame an ordinance that will sufficiently protect the average life and health and still not be so inelastic that it must be enforced without discretion. We must not forget that when we speak of the U. S. as the Land of Freedom, it is seriously near to being a joke.

---

**The Emmanuel Movement** received considerable medical endorsement in the east at its inception a few years ago but, in San Francisco, Rev. Parker Boyd, head of the Emmanuel Health Institute has recently been arrested for diagnosing without a medical license.

---

**Centenarian.** Mrs. Mary Simpson Clingman of Cedarville, Ill., died Oct. 23, aged 106 years and 10 months. Four children survive her, aged from 65 to 74.

---

**Binders for Journals.** We can secure binders for this and other journals of about the same size for about 75 cents apiece, or less if ordered in large quantities. If any of our readers are interested, they are requested to remit and to state size of binder if wanted for other journals.

---

**Poliomyelitis.** A case was reported in Buffalo, Nov. 22—Edward Huntzinger, an infant, 133 Theodore Street.

---

**Morphine and Heroin Seized.** The Federal Inspector confiscated a quantity of these drugs from two negroes at 152 Clinton Street, Buffalo, Nov. 22. Several arrests were made.

